# Predictors of flow diverter stent in large and giant unruptured intracranial aneurysms, single-center experience

**DOI:** 10.1007/s10072-022-06336-w

**Published:** 2022-08-19

**Authors:** Hazem Abdelkhalek, Esam Ahmed Abdelhameed, Ayman Zakarea, Islam El Malky

**Affiliations:** 1 Department of Neuropsychiatry, Tanta University, Tanta, Egypt; 2 Department of Neurosurgery, Tanta University, Tanta, Egypt; 3 Department of Radiology, Kasr Alainy University, Cairo, Egypt; 4 Department of Neurology, South Valley University, Qena, Egypt

**Keywords:** Large aneurysms, Giant aneurysms, Flow-diverter stent, Stent assisted coiling

## Abstract

**Background:**

Flow diversion with or without coiling has been established as the treatment of choice for large unruptured aneurysms. This study aims to assess possible predictors for radiological and clinical outcome such as location of the aneurysm (anterior or posterior circulation), complexity by a branching artery, bifurcation, and adjuvant coiling.

**Methods:**

This study was conducted on 65 consecutive patients with 65 large, unruptured intracranial aneurysms (size ≥ 10 mm) treated with flow diverters. Follow-up angiography was done for 60 patients (92.3%) at 12 ± 8.6 months range from 3 to 36 months.

**Results:**

Complete occlusion was achieved in 50 from 60 aneurysms (83.4%), while 8 aneurysms (13.3%) had neck remnant, and another two aneurysms (3.3%) remained with aneurysmal remnant. Periprocedural complications were encountered in 14 patients (21.5%) with morbidity in six patients (9.2%) and mortality in one patient (1.5%). In a multivariate logistic regression, anterior versus posterior location was less likely associated with worse outcome; adjusted OR (95% CI) of 0.16 (0.07–0.01), *p* = 0.006. Complete occlusion in complex aneurysms with branching artery was 60% versus 88% in simple aneurysms without branching artery (*p*-value = 0.04).

**Conclusions:**

Flow diverter deployment of a large, unruptured aneurysm in the anterior circulation might have a better outcome than one in the posterior circulation. Flow diverter of aneurysms with branching artery or at bifurcation might be associated with aneurysm persistence and complications respectively.

## Introduction

The cumulative rupture rate of posterior circulation untreated large aneurysms and posterior communicating artery aneurysms is as high as 50% in 5 years and slightly lower (40%) in anterior circulation [1]. This high risk can be reduced by early definitive endovascular or surgical treatment. Large and giant aneurysms are a challenge to treat because of the high mortality and morbidity in both endovascular and surgical approaches [2, 3].

Flow diverting stents (FDS) had appeared as a tool for treating these large intracranial aneurysms because they encourage neointimal growth through the neck of the aneurysm leading to the reconstruction of the parent vessel while preserving normal antegrade blood flow. The Food and Drug Administration (FDA) approved the pipeline embolization device (PED) in 2011 as a treatment for adults with wide-necked brain aneurysms > 10 mm in ICA from the petrous to the superior hypophyseal segment after the results of PUFS trial (the Pipeline for Uncoilable or Failed Aneurysms trial) [4]. Then, the FDA expanded the indication to include smaller internal carotid artery aneurysms up to the carotid bifurcation. Nevertheless, some issues such as FDSs for aneurysms in the posterior circulation, bifurcation aneurysms, and the presence of an incorporating branching artery at the neck of the aneurysm are still in a debate [5]. The current study aimed to assess possible predictors for radiological and clinical outcome such as location, presence of an incorporating branching artery, bifurcation, and adjuvant coiling.

## Methods

### Participants

This retrospective study has been conducted based on patients’ records after obtaining the institutional review board approval and according to the Declaration of Helsinki. Sixty-five consecutive patients with unruptured saccular aneurysms larger than 10-mm diameter were collected from June 2016 to June 2021. MRI and MRA brain were performed in all cases to exclude partially thrombosed or dissecting aneurysms. Previously embolized aneurysms were excluded. All aneurysms were confirmed by digital subtraction angiography (DSA). The neck was considered wide if the dome size to neck width ratio was ≤ 1.5. All recruited patients were given two antiplatelet drugs (75 mg/day clopidogrel plus 150 mg/day aspirin) for 10 days before the procedure.

### Endovascular treatment

All procedures were done on Philips biplane (Allura clarity FD 20/15, Netherland) under general anesthesia and through trans-femoral approach. The procedures were performed by two senior neuro-interventionalists with more than 5 years of experience in endovascular therapy. After sheath settlement, injection of heparin was done to preserve the activated clotting time at 2 to 3 times the baseline through the procedure. Next, a proper (6 F) guiding catheter was navigated through the aorta and then positioned at the distal internal carotid artery or vertebral artery.

SILK (Balt, France) or Pipeline (Covidien, USA) FDSs were used in all cases. Multiple FDS were used in 17 aneurysms (26.2%) in an overlapping or telescopic manner according to the operator’s opinion as regards stagnation or position of the first FDS after its deployment. The used microcatheters were Vasco 21 (Balt, France) or marksman (Covidien, USA). A shapeable microwire of 0.14 or 0.16 was used to navigate the microcatheter in the parent artery of the aneurysm. Working projection was selected to visualize the distal and proximal part of the landing area of the flow diverter subsequently; the aneurysms were treated with flow diverter implantation with or without coiling according to the complexity of the anatomy and size of the aneurysm. At the end of the procedure, Vaso-CT was done by the Philips Machine to visualize the opening of the stent and its opposition to the wall of the artery.

Data regarding the aneurysms’ actual size, the width of the aneurysmal neck, aneurysmal type, complex aneurysm with a branching artery at the neck or the dome, and treatment results were collected by two senior neuroradiologists. Raymond’s classification was used to classify the angiographic results of aneurysm occlusion at follow up [6].

Procedural complications were identified. Hemorrhagic complications such as intraparenchymal hemorrhage at the same or other territory and aneurysm rupture up to the last follow-up were reported. Thromboembolic complications such as side branch occlusion, dissections, distal emboli, and thrombus formation inside the stent had been identified. The clinical consequences of these complications were measured by mRS (modified Rankin scale) in the immediate postoperative phase. Clinical deterioration was considered, if mRS increased by one point in comparison to the preoperative score.

All patients were evaluated clinically by two senior neurologists, using mRS at discharge and 3-month post-treatment, either through clinical visits or telephone contact. The post-operative antiplatelet regimen (150 mg aspirin + 75 mg clopidogrel) was administered for 6 months then only 150 mg aspirin for 1 year. Follow-up MRI brain and DSA were performed for all cases at 12 ± 8.6 months, ranging from 3 to 36 months. In-stent stenosis (if ≥ 50% of stent diameter) was recorded at follow-up DSA [7].

### Statistical analysis

The study data was analyzed using SPSS (IBM SPSS Statistics for Windows, Version 24.0. Armonk, NY: IBM Corp). Descriptive statistics were reported as frequencies for categorical data. Median and IQR were used for numerical data which was not normally distributed. Chi-square test, chi-square test for trend, and Fischer’s exact test were used to compare two or more categorical variables. A *p*-value of 0.05 or less was considered statistically significant. Univariate logistic regression and thereafter multivariate logistic regression analysis for variables with *p* < 0.2 were used to find out possible predictors for good outcome regarding mortality, morbidity, and occlusion.

## Results

### Demographic data and aneurysms characteristics

The study included 65 patients who harbored 65 aneurysms. The median age was 55.5 years (IQR: 44.25–62.75 years). Females represented 70.8% of all patients. The initial clinical presentation and vascular risk factors are shown in (Table 1).

**Table 1:** Initial presenting symptoms and risk factors

Clinical presentation	*n* (%)
Headache	40 (61.5%)
Cranial nerve palsy	9 (13.8%)
Motor deficit	7 (10.8%)
Seizures	5 (7.7%)
Visual field affection	4 (6.2%)
Risk factors	*n* (%)
Hypertension	25 (38.5%)
Diabetes mellitus	6 (9.2%)
Smoking	16 (24.6%)
Hyperlipidemia	10 (15.4%)

The median size of aneurysms was 16.4 mm (IQR: 12.50–23.85 mm), ranged from 12 to 39 mm. The median neck width was 7.15 mm (IQR: 5.85–10.24 mm). Aneurysms larger than 25 mm were considered giant aneurysms and their number was 15 aneurysms (23%). Aneurysm characters such as complex aneurysms by an incorporating branching artery, bifurcation aneurysms, neck width, and locations are presented in Table 2.

**Table 2:** Aneurysms data

Aneurysm characters	*n* (%)
Width of the neck	
Narrow necked	54 (83.9%)
Wide necked	11 (16.9%)
Complex aneurysms with an incorporating branching artery at neck	
Complex	11 (16.9%)
Not complex	54 (83.1%)
Bifurcation or sidewall aneurysm	
Bifurcation aneurysm	12 (18.5%)
Side wall aneurysm	53 (81.5%)
Aneurysm location	
Internal carotid artery	51 (78.5%)
Middle cerebral artery	6 (9.2%)
Anterior communicating artery	1 (1.5%)
Basilar tip artery	3 (4.6%)
Basilar trunk artery	2 (3%)
Vertebral artery	1 (1.5%)
Posterior cerebral artery (P1)	1 (1.5%)

### Procedural complications and immediate clinical outcome

**Fig. 1:**
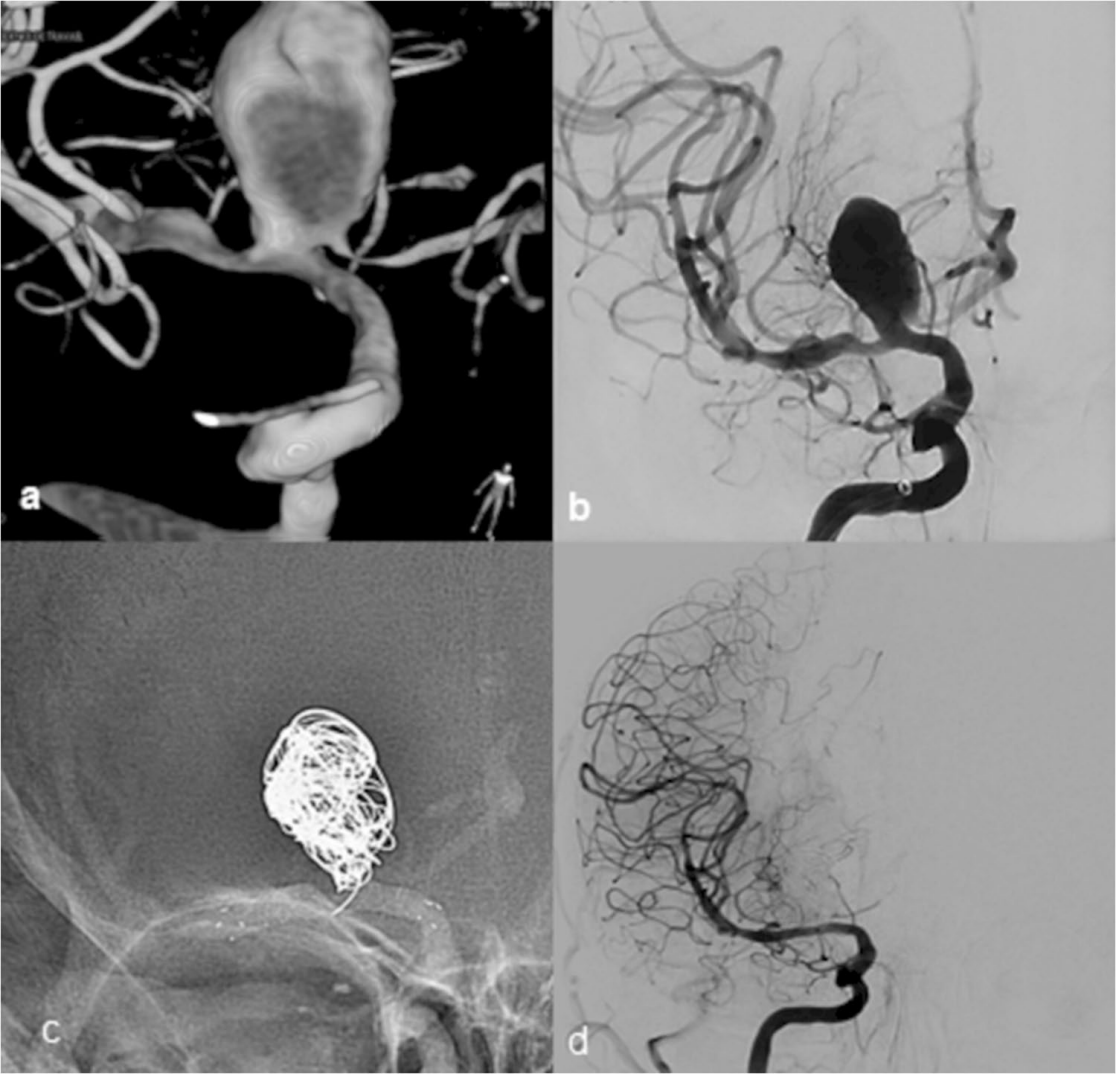
Terminal carotid large (20 mm) aneurysm with coil adjuvant. **a** and **b** 3D and 2D image AP view right ICA showing terminal carotid aneurysm and the right A1 is arising from its sac. **c** Non-subtracted image showing flow diverter and coils. **d** 2D image AP control 12 months showing complete occlusion of the aneurysm

Balloon angioplasty was done in 11 aneurysms (16.9%) due to stenosis in the parent artery or inappropriate apposition of the flow diverter to the arterial wall. Adjuvant coiling was done in 19 aneurysms (29.2%) due to giant size and/or irregular shape (Fig. 1).

Overall procedural complications occurred in 12 aneurysms (18.5%). Thrombus formation occurred inside the stent in three cases and technical problems such as stent dislodgement and improper deployment in three cases leading to deterioration in mRS (modified Ranken scale) in five cases (7.7%). Two cases with side branch occlusion, two cases with dissection, and two cases with distal emboli occurred without clinical deterioration.

Two cases of delayed rupture (3%) occurred 24 h and 3 weeks after the operation. One of them had *mRS* = 5, with an aneurysm at the basilar trunk, and the other one (1.5%) died with an aneurysm complicated with branching PICA (posterior inferior cerebellar artery). The permanent morbidity and mortality rates were 9.2% and 1.5% respectively. Thromboembolic complications occurred in aneurysms at bifurcation more than sidewall aneurysms (41.7% versus 13.2% with *p*-value = 0.04, OR: 4.69 and CI: 1.162–18.962). Complications in large and giant aneurysms occurred in nine and three aneurysms, respectively, without statistical difference (18% against 20%, respectively with *P*-value = 0.9).

Multiple variables regarding clinical outcome such as adjuvant coiling and aneurysm location were tested. It was found that FDS without adjuvant coiling had statistically significant better outcome regarding combined morbidity and mortality (93.5% versus 73.7% with *P*-value = 0.04). FDS at anterior circulation reported better clinical outcome than posterior one (93.1% versus 42.9% with *P*-value = 0.003). In a multivariate logistic regression, the location of aneurysm was the only predictor of clinical outcome (Table 3).

**Table 3:** Multivariate logistic regression of variables affecting clinical outcome

Predictor variable	Unadjusted OR	Adjusted OR	95% CI of OR	*P*-value
Adjuvant coiling	0.2	0.26	0.05–1.48	0.130
Anterior versus posterior circulation	0.06	0.16	0.07–0.01	0.006

*OR* odds ratio, *CI* confidence intervals

### Angiographic follow-up results

**Fig. 2:**
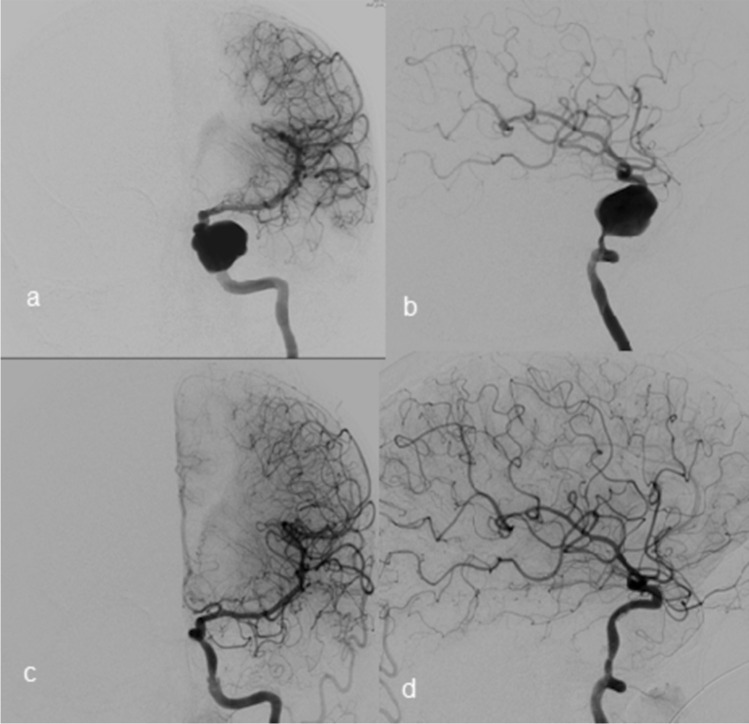
A giant carotid cavernous aneurysm. **a** and **b** DSA (digital subtraction angiography) in AP and lateral views for a giant aneurysm before stenting. **c** and **d** DSA in AP and lateral views after stenting 12-month follow-up

Sixty cases (92.3% of all cases) were followed up at 12 ± 8.6 months, ranging from 3 to 36 months by MRI and DSA. Four patients were lost to follow-up because they were from outside the country and one patient died as previously described. The latest angiographic outcomes reported complete occlusion in 50 patients (83.4%), neck remnant in 8 (13.3%), and sac remnant in two patients (3.3%) (Fig. 2). There was no in-stent stenosis in follow-up imaging.

Complete occlusion in aneurysms with an incorporating branching artery was 60% versus 88% in simple aneurysms without branching artery (*p*-value = 0.04). The presence of a branching artery in the neck of the aneurysm had statistically significant worse occlusion rate but other variables such as aneurysm size of dome (80% versus 60% in large against giant aneurysm, *p*-value = 0.312) or neck width (90.9% versus 79.9% in narrow necked aneurysms against wide necked aneurysms, *p*-value = 0.676), and location of the aneurysms (*p*-value = 0.375) and bifurcation (86.4% versus 75% in sidewall aneurysm against bifurcation one, *p*-value = 0.733) had no effect on occlusion rate.

## Discussion

In our cohort, complete occlusion was achieved in 50 from 60 aneurysms (83.4%), while eight aneurysms (13.3%) had neck remnant, and another two aneurysms (3.3%) remained with sac remnants. Breganti and colleagues reported 85% complete occlusion rate in an Italian multicenter study with 295 aneurysms, using SILK and PED FDS [8]. The PITA trial (The Pipeline Embolization Device for the Intracranial Treatment of Aneurysm Trial) reported 93.2% complete occlusion rate in 6-month follow-up without in-stent stenosis but dome size and neck size were less than the current study (mean dome size = 11.5 mm and mean neck size = 9.07 mm) [9]. In this study, there was no recurrence or rebleeding after confirmation of complete occlusion such as in PUFS 5-year results [10]. In a multicenter study (four Spanish hospitals), the complete occlusion was 78.1% after 1-year follow-up without recurrence or retreatment but in-stent stenosis was 6.3% [11]. ASPIRe was a prospective, single-arm, multi-center registry for 191 patients undergoing PED treatment of 207 intracranial aneurysms (anterior and posterior circulations); complete occlusion rate was 78.6% in 6 months [12].

In the present study, complete occlusion in complex aneurysms with branching artery was 60% versus 88% in simple aneurysms without branching artery (*p*-value = 0.04) but without statistically significant difference on morbidity. Multiple studies reported persistence of aneurysm filling in complex aneurysms with branching artery because FDS does not close this branch due to continuous blood demand from the branch’s territory, and so, the filling of the aneurysm will continue [13, 14]. We did not find a relation between occlusion rate and neck width or size of the aneurysms, which was similar to other studies [15–17], but other studies reported this relation because they included a high percentage of small-sized aneurysms [18].

The study procedural complication rate was 18.5%, resulting in combined mortality and mortality 10.7% which was comparable with many FDS studies. Zhou G. and colleagues reported in a meta-analysis the overall complication rate was 14.6%, with more complications in the posterior circulations than the anterior one (44.7% versus 23.7%) which is similar to the study results [19]. It could be due to many perforators at the vertebrobasilar system.

Hemorrhagic complications are the most devastating ones and associated with worse clinical outcomes in FDS. The present study did not have intraparenchymal hemorrhage (IPH) but it had delayed rupture aneurysms (two aneurysms = 3%) which comparing to different previous results. In two large meta-analyses, the calculated IPH and aneurysm rupture rates were (2.9, 2.9%) and (1.8, 2%) respectively, which were higher in giant aneurysms [19, 20]. The IntrePED study showed similar results, with IPH and aneurysm rupture rates of 5.8% and 5.8%, respectively [21]. Almandoz and colleagues reported a relation between P2Y12 reaction unit values and all major perioperative thromboembolic and hemorrhagic complications after PED procedures [22]. Furthermore, the hemorrhagic transformation from ischemic stroke, dual antiplatelet therapy, and hemodynamic changes from flow diverter placement may contribute to IPH [23].

In the IntrePED study, the rate of thromboembolic complications was increased in large or giant aneurysms because of intra-aneurysmal thrombosis or a prolonged procedure time, ranging from 5.2 to 13.5% [21]. It was found more thromboembolic complications at bifurcation aneurysms which were similar to other studies [24] and contradictory to others [25]. The contradictory results might be due to the treatment of small-sized aneurysms in these studies in contrast to the current study which included only large and giant aneurysms.

The morbidity and mortality rates of the study were 9.2% and 1.5%, respectively, comparing with other multiple studies. Ye G. and colleagues reported in a meta-analysis that the total morbidity and mortality rates are 9.8 and 3.8%, respectively [20]. The location of the aneurysm was a risk factor for morbidity and mortality in this study that was reported in many studies [26, 27]. In FIAT (The Flow Diversion in the Treatment of Intracranial Aneurysm Trial) which is the only randomized controlled study, morbidity and mortality were lower for proximal carotid aneurysms (8.0%) than for posterior circulation aneurysms (46.2%) [28]. The current study revealed that adjuvant coiling associated with more morbidity and mortality which is statistically significant but not a predictor according to multivariate logistic regression. Zhou and colleagues reported adjuvant coiling as a predictor for occlusion rate and clinical outcome [29]. Bad outcome with adjuvant coiling might be due to more prolonged procedure and the use of more devices that might lead to more thromboembolic complications.

The study had some limitations such as the retrospective design with all intrinsic bias of the study design such as selection bias. One of the most important limitations of this study is the relatively low number of cases which could affect the results in terms of limiting the statistical power of identifying potentially significant variables associated with complications and outcome. The study was performed in a single center. The interventional society may need more RCTs on FDS in certain situations such as bifurcations, complex aneurysms with branching artery, and adjuvant coiling.

## Conclusions

Flow diverter deployment of a large unruptured aneurysm in the anterior circulation might have better outcome than this one in the posterior circulation. Flow-diverter stent in aneurysms with an incorporating branching artery or at bifurcation might be associated with aneurysm persistence and complications respectively.

## References

[1] Wiebers DO, Investigators ISoUIA (2003). Unruptured intracranial aneurysms: natural history, clinical outcome, and risks of surgical and endovascular treatment. The Lancet..

[2] Sluzewski M, Menovsky T, Van Rooij WJ, Wijnalda D (2003). Coiling of very large or giant cerebral aneurysms: long-term clinical and serial angiographic results. Am J Neuroradiol.

[3] Jafar JJ, Russell SM, Woo HH (2002). Treatment of giant intracranial aneurysms with saphenous vein extracranial-to-intracranial bypass grafting: indications, operative technique, and results in 29 patients. Neurosurgery.

[4] Becske T, Kallmes DF, Saatci I, McDougall CG, Szikora I, Lanzino G (2013). Pipeline for uncoilable or failed aneurysms: results from a multicenter clinical trial. Radiology.

[5] Bonney PA, Connor M, Fujii T, Singh P, Koch MJ, Stapleton CJ (2020). Failure of flow diverter therapy: predictors and management strategies. Neurosurgery..

[6] Roy D, Milot G, Raymond J (2001). Endovascular treatment of unruptured aneurysms. Stroke.

[7] Kokkinos J, Strong J, Brown M, Levine S, Silliman S, Pessin M (1995). Warfarin-aspirin symptomatic intracranial disease study group. The warfarin-aspirin symptomatic intracranial disease study. Neurology..

[8] Briganti F, Napoli M, Tortora F, Solari D, Bergui M, Boccardi E (2012). Italian multicenter experience with flow-diverter devices for intracranial unruptured aneurysm treatment with periprocedural complications—a retrospective data analysis. Neuroradiology.

[9] Nelson P, Lylyk P, Szikora I, Wetzel S, Wanke I, Fiorella D (2011). The pipeline embolization device for the intracranial treatment of aneurysms trial. Am J Neuroradiol.

[10] Becske T, Brinjikji W, Potts MB, Kallmes DF, Shapiro M, Moran CJ (2017). Long-Term clinical and angiographic outcomes following pipeline embolization device treatment of complex internal carotid artery aneurysms: five-year results of the pipeline for uncoilable or failed aneurysms trial. Neurosurgery.

[11] Pumar JM, Banguero A, Cuellar H, Guimaraens L, Masso J, Miralbes S (2017). Treatment of intracranial aneurysms with the SILK embolization device in a multicenter study. A retrospective data analysis Neurosurgery.

[12] Kallmes DF, Brinjikji W, Boccardi E, Ciceri E, Diaz O, Tawk R, Woo H, Jabbour P, Albuquerque F, Chapot R, Bonafe A, Dashti SR, Delgado Almandoz JE, Given II C, Kelly ME, Cross III DT, Duckwiler G, Razack N, Powers CJ, Fischer S, Lopes D, Harrigan MR, Huddle D, Turner IV R, Zaidat OO, Defreyne L, Mendes Pereira V, Cekirge S, Fiorella D, Hanel RA, Lylyk P, McDougall C, Siddiqui A, Szikora I, Levy E (2016). Aneurysm Study of Pipeline in an Observational Registry (ASPIRe). Interv Neurol..

[13] Wallace AN, Kayan Y, Austin MJ, Almandoz JE, Kamran M, Cross DT, Moran CJ, Osbun JW, Kansagra AP (2017). Pipeline embolization of posterior communicating artery aneurysms associated with a fetal origin posterior cerebral artery. Clin Neurol Neurosurg.

[14] Chiu AHCA, Wenderoth JD, De Villiers L, Rice H, Phatouros CC (2015). Long-term follow-up results following elective treatment of unruptured intracranial aneurysms with the pipeline embolization device. AJNR Am J Neuroradiol.

[15] Mut F, Raschi M, Scrivano E, Bleise C, Chudyk J, Ceratto R (2015). Association between hemodynamic conditions and occlusion times after flow diversion in cerebral aneurysms. Journal of NeuroInterventional Surgery.

[16] Brinjikji W, Murad MH, Lanzino G, Cloft HJ, Kallmes DF (2013). Endovascular treatment of intracranial aneurysms with flow diverters. Stroke.

[17] Saatci I, Yavuz K, Ozer C, Geyik S, Cekirge HS (2012) Treatment of intracranial aneurysms using the Pipeline flow-diverter embolization device: a single-center experience with longterm follow-up results. AJNR Am J Neuroradiol, 1436–46 10.3174/ajnr.A3246 PMC7966552 22821921

[18] Bender MT, Colby GP, Lin L-M, Jiang B, Westbroek EM, Xu R (2018). Predictors of cerebral aneurysm persistence and occlusion after flow diversion: a single-institution series of 445 cases with angiographic follow-up. J Neurosurg JNS.

[19] Zhou G, Su M, Yin Y-L, Li M-H (2017). Complications associated with the use of flow-diverting devices for cerebral aneurysms: a systematic review and meta-analysis. Neurosurgical Focus FOC.

[20] Ye G, Zhang M, Deng L, Chen X, Wang Y (2016). Meta-analysis of the efficiency and prognosis of intracranial aneurysm treated with flow diverter devices. J Mol Neurosci.

[21] Kallmes DF, Hanel R, Lopes D, Boccardi E, Bonafé A, Cekirge S (2015). International retrospective study of the pipeline embolization device: a multicenter aneurysm treatment study. AJNR Am J Neuroradiol.

[22] Delgado Almandoz JE, Crandall BM, Scholz JM, Fease JL, Anderson RE, Kadkhodayan Y (2013). Pre-procedure P2Y12 reaction units value predicts perioperative thromboembolic and hemorrhagic complications in patients with cerebral aneurysms treated with the pipeline embolization device. J Neurointerv Surg..

[23] Cruz JP, Chow M, O'Kelly C, Marotta B, Spears J, Montanera W (2012). Delayed ipsilateral parenchymal hemorrhage following flow diversion for the treatment of anterior circulation aneurysms. AJNR Am J Neuroradiol.

[24] Caroff J NH, Mihalea C, D’Argento F, Khalek HA, Ikka L, et al. Flow-diverter stents for the treatment of saccular middle cerebral artery bifurcation aneurysms. 2016;37(2):279-84 10.3174/ajnr.A4540 PMC7959957 26405085

[25] Yavuz K, Geyik S, Saatci I, Cekirge HS (2014). Endovascular treatment of middle cerebral artery aneurysms with flow modification with the use of the pipeline embolization device. AJNR Am J Neuroradiol.

[26] Chalouhi N, Tjoumakaris S, Dumont AS, Gonzalez LF, Randazzo C, Starke RM (2013). Treatment of posterior circulation aneurysms with the pipeline embolization device. Neurosurgery.

[27] Siddiqui AH, Abla AA, Kan P, Dumont TM, Jahshan S, Britz GW (2012). Panacea or problem: flow diverters in the treatment of symptomatic large or giant fusiform vertebrobasilar aneurysms. J Neurosurg.

[28] Raymond J, Gentric JC, Darsaut TE, Iancu D, Chagnon M, Weill A (2017). Flow diversion in the treatment of aneurysms: a randomized care trial and registry. J Neurosurg.

[29] Zhou Y, Wu X, Tian Z, Yang X, Mu S (2020). Pipeline embolization device with adjunctive coils for the treatment of unruptured large or giant vertebrobasilar aneurysms: a single-center experience. Front Neurol.

